# A comparative observational study on intrasphincteric injections with Botulinum toxin vs. enteral neuromodulation in chronic refractory constipation

**DOI:** 10.1186/s12887-023-04217-5

**Published:** 2023-09-08

**Authors:** Mona Walther, Hanna Müller, Christel Weiß, Roman Carbon, Sonja Diez, Manuel Besendörfer

**Affiliations:** 1 Department of Surgery, Section of Pediatric Surgery, Friedrich-Alexander-Universität Erlangen-Nürnberg (FAU), University Hospital Erlangen, Loschgestrasse 15, 91054 Erlangen, Germany; 2 Neonatology and Pediatric Intensive Care, Department of Pediatrics, University Hospital Marburg, 35043 Baldingerstraße, Marburg, Germany; 3 Department of Medical Statistics, Biomathematics, and Information Processing, Medical Faculty, Mannheim of Heidelberg University, Theodor-Kutzer-Ufer 1-3, 68167 Mannheim, Germany

**Keywords:** Botulinum toxin, Chronic refractory constipation, Fecal incontinence, Hirschsprung disease, Enteral neuromodulation.

## Abstract

**Purpose:**

Botulinum toxin injections in the anal sphincter apparatus (Botox) and enteral neuromodulation (ENM) are options for treatment of refractory chronic constipation. We present a retrospective comparative observational study.

**Patients and methods:**

From 2014 to 2022, pediatric patients with chronic constipation were either treated with Botox or ENM with continuation of conservative treatment. Comparison was conducted regarding the primary outcome variables defecation frequency, stool consistency, and abdominal pain. Secondary outcomes were fecal incontinence, enuresis, change of medication and safety of treatment.

**Results:**

19 Botox patients (10 boys, 9 girls, 12 patients with Hirschsprung disease (HD), 7 patients with functional constipation (FC)) were compared to 24 ENM patients (18 boys, 6 girls, 12 HD patients, 7 FC patients). Groups differed significantly in age (5.0 years (Botulinum toxin) and 6.5 years (ENM), mean values, p-value 0.008). Improvement of constipation was seen in 68% (n = 13/19) of Botox and 88% (n = 21/24) of ENM patients (p = 0.153). Influence of etiology on therapeutic effects was not observed. Complications were minor.

**Conclusions:**

Botox and ENM can be considered as valuable and effective treatment options in refractory chronic constipation. Prospective, large-population studies should be designed to enable improved evidence.

## Introduction

Childhood constipation is a frequent diagnosis presenting with a variability of heterogeneous symptoms, which can result from pathophysiological defects in the anal sphincters and/or colonic motility [1]. A common underlying cause is functional constipation (FC) with a prevalence of about 29.6% worldwide, combining rectal evacuation disorders and gastrointestinal motility dysfunctions [2]. Furthermore, symptoms are seen in post-surgical patients: e.g., patients with Hirschsprung disease (HD) are affected in 8–30% according to recent reports [3]. These numbers justify the need for therapeutic options and the increased ambition for introducing new therapeutic possibilities.

This need led to the implementation of Botulinum toxin injections in the anal sphincter apparatus in 1997 (Botox injections) [4–6] and sacral neuromodulation in 1995 (SNM) [7]. Both approaches present less invasive and more reversible interventions compared to conventional surgical options. Botox antagonizes the overactivity of the anal sphincter apparatus which is seen as one of the main causes of chronic constipation [8, 9]. The reduction of the sphincter tone supports conventional options (e.g., laxants and irrigations) in symptom control [10, 11]. HD patients were the first being treated with Botox injections to alleviate obstructive symptoms [4]. Since then, the application was expanded to other defecation disorders in pediatric patients [5, 6, 12]. Keshtgar et al. reported on improved defecation frequency and less encopresis one year after treatment with Botox in patients with functional constipation [9]. Furthermore, Roorda et al. confirmed the efficacy of Botox in a meta-analysis of HD patients with persisting constipation after surgical repair (66% with reduction of symptoms) [13].

SNM, a surgically implanted pacemaker to stimulate sacral nerval roots S3/4, was adapted for treatment of adult fecal incontinence > 20 years ago [14]. Variables of invasive SNM have been transferred to a non-invasive option for children and adolescents, creating a transabdominal electric field via cutaneous, attachable electrodes for stimulation, called enteral neuromodulation (ENM). The continuous, low frequency electrical stimulation improves mobility and perfusion of the gastrointestinal tract [15] and might show an additional effect on the neuroplasticity of the enteric nerves [16]. Effects of ENM on constipation in HD and FC patients could recently be confirmed in a randomized controlled trial [17]. However, mechanisms of action are not fully elucidated, while its influence on sacral spinal nerves and - in comparison to the classical SNM – its influence enlarged to the enteric nervous system are discussed, leading to effects in both, colon motility and sphincter regulation [18].

We are here presenting the first retrospective study to compare these two potent, yet less invasive surgical options in the treatment of chronic constipation in childhood and adolescence. As treatment with Botox was not effective in 100% of patients, included the risks of general anesthesia and needed in some cases repetitive surgery, Botox injections were replaced by the implementation of ENM in 2018 in our specialized center, which led to the presented study to assess the justification of this replacement.

## Patients and methods

This retrospective single center study analyses patients with chronic constipation from 2014 to 2022 in a comparative design. It comprises the time periods of 2014–2018 for the application of Botulinum toxin (Botox) and of 2018–2022 for the application of enteral SNM (ENM).

The study was approved by the local ethics committee in accordance with the declaration of Helsinki (1964) and its later amendments (No. B18_20). The need for informed consent was waived by the Ethikkommission der Friedrich-Alexander-Universität Erlangen-Nürnberg (FAU) due to the nature of the study.

### Eligibility criteria

Data were collected from medical reports of all patients who were treated within our center with either Botulinum toxin or ENM. As the application of ENM has been conducted within a clinical trial since 2018, specialized questionnaires were additionally used in this subgroup to collect data on symptoms and change of symptoms during therapy, including evaluation of the Bristol Stool Scale [19].

Patients were chosen for participation if they met the following inclusion criteria:

- 0–17 years of age.
- diagnosis of Hirschsprung disease (HD, pre- or postsurgical) or of functional constipation with symptomatic chronic constipation according to the ROME IV criteria [20] for more than 3 months with or without fecal incontinence (irrespective of primary sphincter dysfunction or suggested overflow-incontinence).
- in cases of Hirschsprung disease: confirmation of diagnosis via rectal biopsies.
- exclusion of metabolic/inflammatory/neuronal causes or mechanical obstruction of chronic constipation.

### Therapeutic techniques

#### Botulinumtoxin group (Botox)

Botox injections took place under general anaesthesia. A usual dose of 50 IE (range 20–60 IE) was applied to four quadrants of the anal sphincter and was applied age- and weight-adjusted. Localization of the injections were at 3, 6, 9 and 12 o´clock in the lithotomy position. Patients received 1 to 4 injections in total. The internal anal sphincter was identified via manual palpation.

#### Enteral neuromodulation group (ENM)

**Fig. 1:**
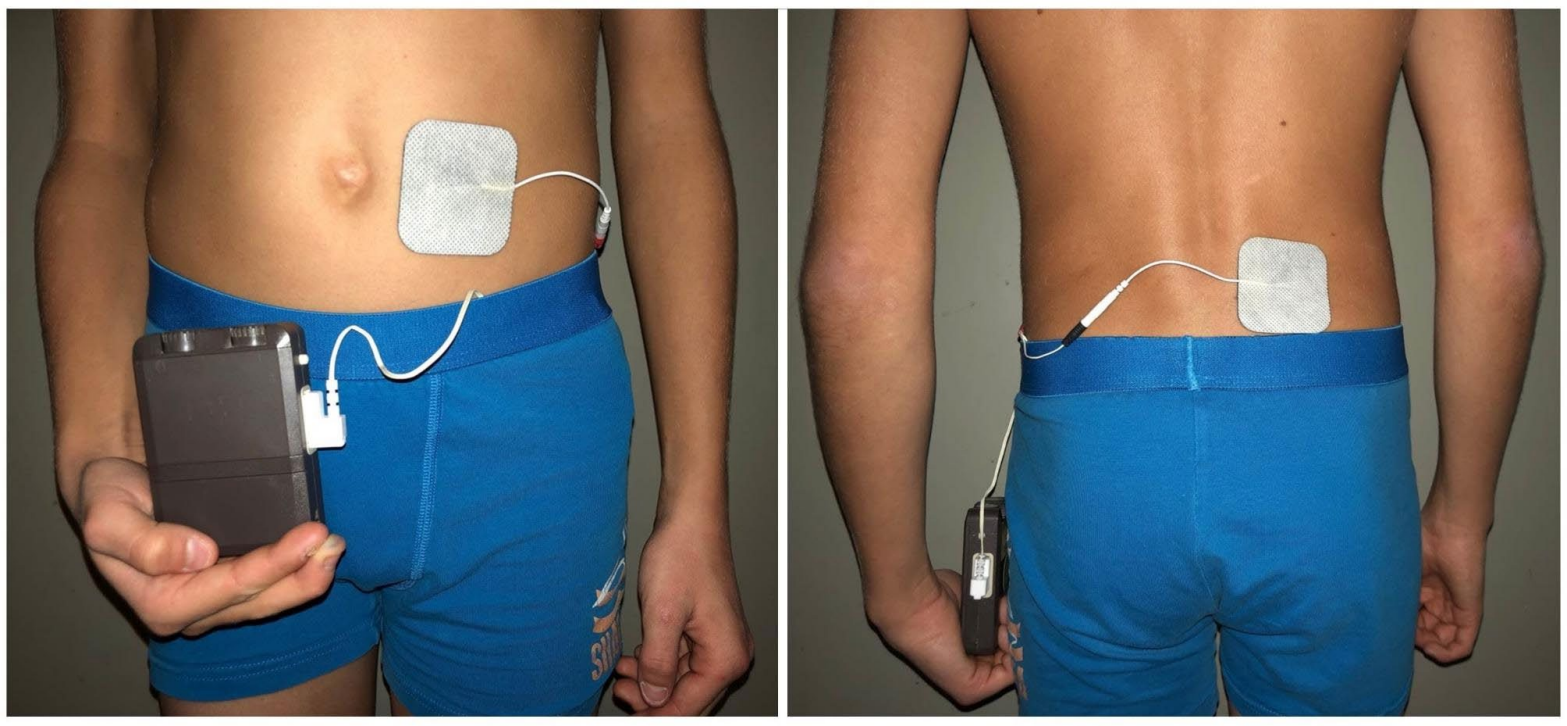
Placement of the attachable electrodes of enteral neuromodulation

The non-invasive variant of sacral neuromodulation was administered via two cutaneously adhesive electrodes [Stimex R -electrodes, Pierenkemper GmbH, 35,630 Ehringshausen (pierenkemper.eu)], placed paravertebrally and periumbilically (Fig. 1). Detailed stimulation techniques have been described previously [18] and are administered via a pulse generator (Ostimex® ProfiPlus TENS/EMS 335,035) [17], which is preset for voltage, pulse width and frequency prior to therapy. Stimulation intensity was individually set by each patient to achieve a comfortable stimulation below the pain threshold. ENM was continuously applied for 12 weeks in each patient.

### Study design

The two groups were compared regarding the following outcome variables: defecation frequency, stool consistency, and abdominal pain. A clinically relevant improvement was defined in cases with at least 2/3 fulfilled criteria, achieving symptom control. Additionally, fecal incontinence, enuresis, and change of medication during treatment as secondary outcome variables were assessed. Adverse events were collected individually for both study groups.

### Statistical analysis

All statistical calculations were performed using the SAS software (release 9.4; SAS Institute Inc., Cary, NC, USA). Clinical factors in correlation with effectiveness of the therapy were compared using 2 independent sample t-tests for continuous and normally distributed data and Mann-Whitney-U-tests for continuous, non-normally distributed data, and chi-square and Fisher’s exact tests for categorial, non-normally distributed data. When analyzing multiple factors, multivariate analysis was not performed due to the small number of patients. Results were considered to be statistically significant at p < 0.05.

## Results

**Fig. 2:**
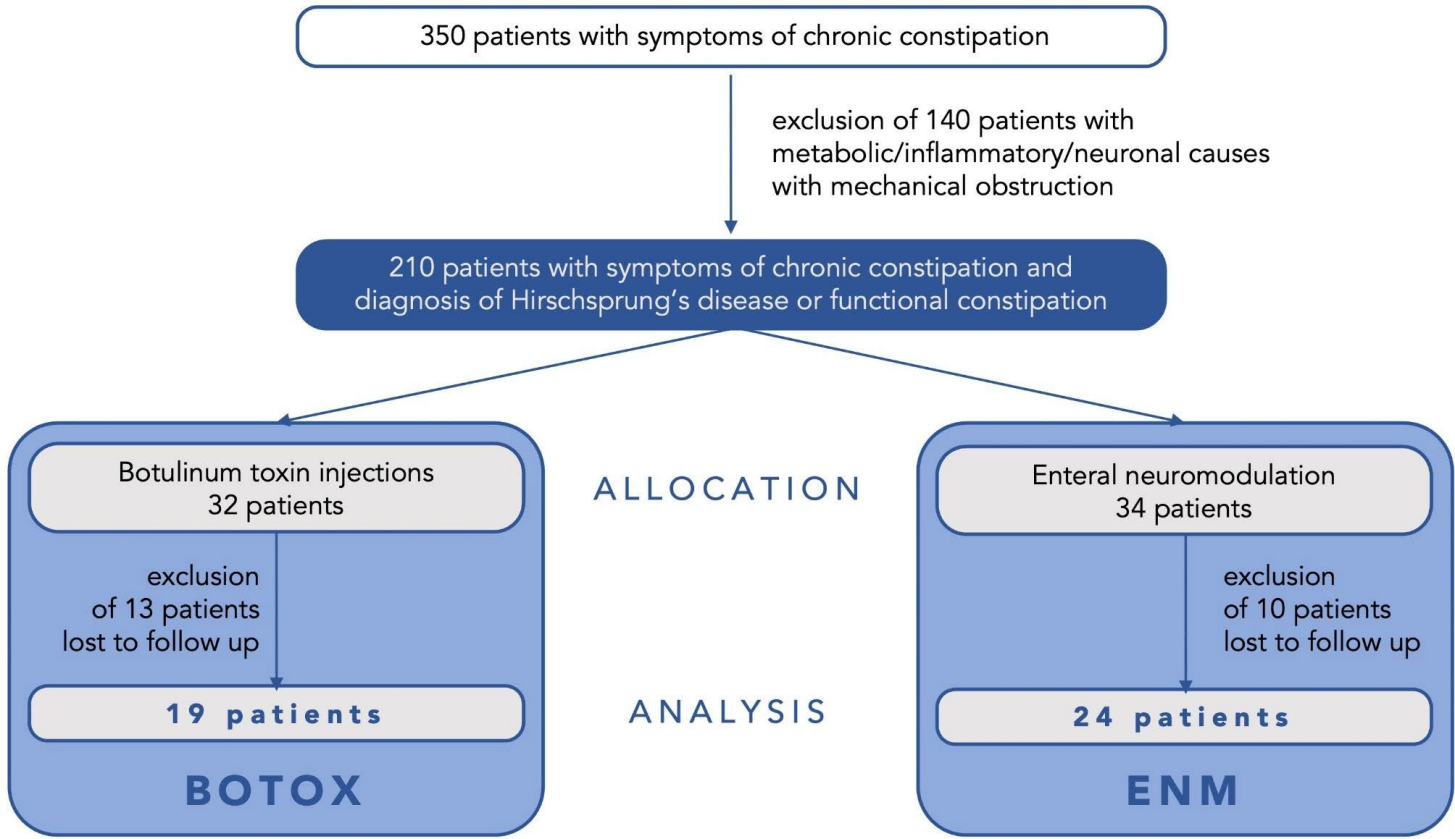
Study design of the retrospective study

Over the study period of 9 years, we treated a total of 32 patients with chronic constipation with Botox injections and a total of 34 patients by adjusting ENM, respectively. After exclusion of 23 patients lost to follow up or due to premature termination of therapy, analyses were conducted for 19 Botox- and 24 ENM-patients. Premature termination of therapy included only ENM patients. In these, application of treatment for at least 8 h/day was not possible. The study’s design is illustrated in Fig. 2.

Baseline demographics of the included patients are shown in Table 1. There was a significant difference between the groups in terms of mean age (5.0 years in the Botox group and 6.5 years in the ENM group, p-value 0.008). Differentiation between colonic hypomotility or dysfunctions of the sphincter was not sufficiently possible based on medical reports.

**Table 1:** Baseline demographic data of study population

	ENM (n = 24)		Botox (n=19)		p-value
Age [mean in years (range)]	6.5 (2–16)		5.0 (0–11)		**0.008**
Sex [n (%)]					0.126
Female	6	25%	9	47%	
Male	18	75%	10	53%	
Diagnosis [n (%)]					0.388
Chronic constipation	12	50%	7	37%	
Hirschsprung’s disease	12	50%	12	63%	
Rectal surgical interventions prior to study [n (%)]					0.257
Yes	7	29%	2	11%	
No	17	71%	17	89%	
Medication [n (%)]					**< 0.001**
Oral (PEG, Prucaloprid, Lactulose)	19	80%	4	21%	
Rectal (saline/non-saline enema, Lecicarbon)	2	8%	0	0%	
Both	1	4%	15	79%	
None	2	8%	0	0%	
Preexisting fecal incontinence [n (%)]					**0.012**
Yes	18	75%	7	37%	
No	6	25%	12	63%	
Preexisting urinary incontinence [n (%)]					0.553
Yes	11	46%	7	37%	
No	13	54%	12	63%	

Treatment options included lifestyle changes and intensified toilet training in all cases before treatment. Additional treatment with oral or rectal medication remained unchanged during treatment with Botox or ENM.

Patients treated with Botox received 1–4 injections (median 1 injection) based on the methods described above. 7 patients received more than one injection within a time period of 25 weeks (median, range 12–160 weeks). Best possible therapeutic response was then evaluated. Only in one of these 7 patients, no treatment success was observable.

Neuromodulation therapy was continued for 12 weeks in each patient. The duration of stimulation was conducted for at least 8 h per day (range 8–24 h). Stimulation intensity ranged from 2 to 8 mA (median 6.0 mA).

A clinically relevant improvement (defined as ≥ 2 fulfilled primary outcome variables) was seen in 68% of Botox patients, compared to 88% of ENM patients (p = 0.153). This reflects numbers of subjective therapeutic success, as collected in the study’s participants (ENM 18/24 (75%) vs. Botox 13/19 (68%)). There was no significant difference between both study groups regarding primary and secondary outcome variables. The comparison of results is summarized in Table 2. Complications of the therapy methods were rarely seen: In the Botox group, increased fecal incontinence was reported in one patient who had already suffered from this condition prior to treatment. In the ENM group, aggravation of urinary incontinence was noted in 2 patients with previously diagnosed enuresis. Additional 4 patients suffered from cutaneous irritation due to attached electrodes, which was successfully treated with the change to hypoallergenic electrodes. In no case, side effects led to a premature termination of therapy.

**Table 2:** Overall primary and secondary outcome variables

Clinically significant improvement compared to baseline in	ENM (n = 24)		Botox (n = 19)		p-value
Overall responsiveness (≥ 2 fulfilled primary outcome variables) [n (%)]	21	88%	13	68%	0.153
Defecation frequency [n (%)]	13	54%	13	68%	0.342
Defecation consistency [n (%)]	14	58%	13	68%	0.497
Abdominal pain [n (%)]	17	71%	13	68%	0.864
Fecal incontinence [n (%)]	14/18	78%	6/7	86%	0.633
Urinary incontinence [n (%)]	3/11	30%	3/7	75%	0.665
Change of medication [n (%)]	12	50%	9	47%	0.864

We further analyzed potential associations of outcome regarding etiology of symptoms (classified in patients with HD and patients with FC, see Table 3). No difference concerning outcome variables could be seen.

**Table 3:** Primary and secondary outcome variables, associated with diagnosis of Hirschsprung disease (HD) and functional constipation (FC).

Clinically significant improvement compared to baseline in	ENM (n = 24)		Botox (n = 19)		p-value
Concerning diagnosis	HD (n = 12)	FC (n = 12)	HD (n = 12)	FC (n = 7)	
Overall responsiveness (≥ 2 fulfilled primary outcome variables) [n (%)]	10 83%	11 92%	8 67%	5 71%	0.495
Defecation frequency [n (%)]	6 50%	7 58%	8 67%	5 71%	0.841
Defecation consistency [n (%)]	8 67%	6 50%	8 67%	5 71%	0.795
Abdominal pain [n (%)]	7 58%	10 83%	8 67%	5 71%	0.604
Fecal incontinence [n (%)]	8/11 73%	6/9 67%	4/5 80%	2/2 100%	1.000
Urinary incontinence [n (%)]	1/4 25%	2/9 22%	2/3 67%	1/4 25%	0.606
Change of medication [n (%)]	6 50%	6 50%	5 42%	4 57%	0.974

## Discussion

Alongside established conventional and surgical options in the treatment of chronic constipation irrespective its etiology, we are working on innovative options for refractory patients in our specialized pediatric surgery center with coloproctological focus. With their minimally invasive character, both Botox injections and ENM are adding valuable options [9, 17] and have, to our knowledge, not been compared regarding efficacy so far.

Based on an observational comparative study, we can state the following results:

1. We observed an overall efficacy of 68% in Botox patients and 88% in ENM patients. Based on the small population, we failed to reach significance in the comparison of the two approaches. Although we failed to reach significant conclusions based on this data, we observed a superior subjective patient’s satisfaction with ENM therapy compared to Botox injections.
2. Although we accept limitations of a small population with consecutive statistical restrictions, therapeutic efficacy could not be seen in association with etiology.
3. Both options are only tested in combination with the continuation of conservative approaches and seem to function as powerful add-on options in established treatment algorithms.
4. Side effects are rare. However, one patient presenting with an aggravation of fecal incontinence estimated fecal incontinence under Botox therapy as more troublesome compared to the individual estimation of patients affected with side effects of ENM. Most importantly, urinary and cutaneous side effects under ENM were easily to treat and had not to be endured until effects of Botox faded. However, this subjective assessment has to be evaluated in enlarged population-based studies.

We suggest to discuss indication for therapeutic options individually depending on patient’s age, as the presented subgroups differed significantly in age. Botox is proposed to be a supportive treatment option especially in younger children who undergo toilet training [11].

We support the hypothesis that the most valuable aspect of Botox might be the facilitation of an independence in the process of defecation regulation without disturbance of other visible therapies. This is additionally highlighted by the fact that there is a substantial improvement of fecal incontinence in patients being treated with Botox. Botox seems to interrupt a vicious cycle of overflow-incontinence and restrictive bowel movements. Nevertheless, loss of efficacy and the necessity of surgical setting should be considered. In older and/or more traumatized children, on the other hand, ENM might be more favorable as rectal manipulation can be avoided. Furthermore, this therapy is precepted as a therapy of autonomy: understanding, settings and intensity can be mainly set by the patients themselves.

There are challenges based on the study’s design, leading to limitations to the presented study.

The retrospective character and the long observational period might cause differences regarding indications for treatment and additional conservative or surgical treatment modalities. This is primarily reflected by the application of enemas, which was reduced within the observational period and therefore involves almost exclusively Botox patients. However, an improved tolerance towards treatment with enema as rectal procedures due to the younger age and the relaxation of the sphincter tone to reduce pain and discomfort, has to be discussed.

The study does moreover not include an evaluation of possible interactions of oral or rectal medication with the proposed two therapeutic approaches. These interactions might have a substantial influence on therapeutic efficacy and should be assessed in a prospective study design with an enlarged population.

Furthermore, preexisting fecal incontinence was seen more frequently in ENM patients. That raises the question if these patients were not regarded as suitable for Botox injections beforehand in order to avoid the risk of fecal incontinence as the major adverse event of Botox injections. However, also fecal incontinence improved in several patients with Botox injections. Side effects might therefore be biased.

The small population of the study, the lack of validated outcome scores, and the relevant percentage of patients lost to follow up in both groups additionally limit the study’s value. Conclusions should therefore be drawn cautiously.

Conclusively, we propose to discuss indications for both options carefully and individually regarding side effects and age. A replacement of one option might accordingly not be justified. Botox injections and most modern approaches like ENM should be regarded as valuable and effective treatment adjunctions in chronic constipation, irrespective of the underlying etiology. Further prospective, randomized interventional case-control studies are needed to investigate adequate therapy strategies for chronic constipation in children at our institution.

## Data Availability

The dataset supporting the conclusions of this article is included within the article. Competing interests.

## Abbreviations

Botox: Botulinum toxin
FC: Functional constipation
HD: Hirschsprung disease
ENM: Enteral neuromodulation
PEG: Polyethylenglycol
SNM: Sacral neuromodulation
